# Cyclophilin D at the Heart of Neonatal Hypoxia

**DOI:** 10.1016/j.jacbts.2026.101570

**Published:** 2026-05-26

**Authors:** Joseph W. Gordon

**Affiliations:** Department of Biomedical Sciences, Faculties of Medicine and Veterinary Medicine, University of Prince Edward Island, Charlottetown, Prince Edward Island, Canada; Department of Pediatrics and Child Health, Rady Faculty of Health Sciences, University of Manitoba, Winnipeg, Manitoba, Canada; Children’s Hospital Research Institute of Manitoba, Winnipeg, Manitoba, Canada

**Keywords:** cardiac development, cardiomyocyte maturation, cyclosporin A, mitochondria, NIM811

**Figure:**
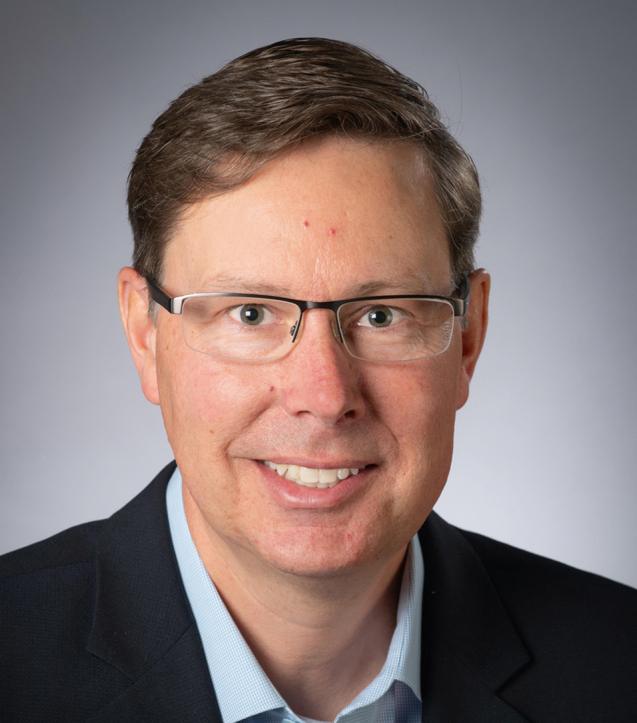

Mitochondrial permeability transition has long been associated with regulated or “programmed” cell death. Seminal work in 2005 demonstrated that the mitochondrial matrix protein cyclophilin D was a critical regulator of mitochondrial permeability transition, where genetic deletion of cyclophilin D desensitized cells to triggers of necrosis (ie, calcium and oxidative stress), but not classical inducers of apoptosis.^1^^,^^2^ In addition, cyclophilin D deletion was able to reduce cardiac infarct size following ischemia-reperfusion in a mouse model.^2^ These exciting observations changed how scientists thought about cell death in general, but also set the stage for a field of research focused on the regulation of necrotic cell death in pathological remodeling, most notably in cardiac-related diseases.^3^

However, how these regulated necrotic pathways were selected for throughout evolution, and whether they play a role in mammalian development remained largely unknown. This is an important biological consideration, because pathological mechanisms often “highjack” or commandeer embryonic or developmental pathways as either a driver of, or a compensation for, the disease state. In 2005, the development role for apoptosis was clearly established and became a critical aspect of our definition of “programmed cell death.” Yet, the concept of regulated necrosis was new and the developmental role of mitochondrial permeability transition unexplored. This changed in 2011, when George A. Porter’s laboratory demonstrated a developmental role for mitochondrial permeability transition pore (PTP) closure in the embryonic heart.^4^ Using a mouse model of embryonic development, the Porter laboratory showed that permeability transition decreases around E13.5 resulting in cardiac mitochondrial maturation, with a more fused morphology, decreased ROS production, and increased mitochondrial membrane potential.^4^ All of these are important for the accumulation of mitochondria in cardiac myocytes and needed for postnatal life. Moreover, this maturation phenotype could be accelerated in E9.5 cardiac myocytes by genetic deletion of, or pharmacological inhibition of, cyclophilin D.^4^ Perhaps most intriguing, the acceleration of mitochondrial maturation by PTP closure increased the differentiation state of the myocyte contractile apparatus.^4^ These findings place PTP closure upstream of changes in mitochondrial morphology, mitochondrial function, and myocyte differentiation, and provide a critical development role permeability transition in cardiac development. I was privileged to be a new investigator around this time, and the work of Dr Porter greatly influenced my early career. My laboratory continued this line of investigation and showed that the cardiac-enriched transcriptional coactivator myocardin can regulate PTP closure during development through a mechanism contingent on the BCL-2 family member Nix (ie, BNIP3L).^5^

In this issue of *JACC: Basic to Translational Science*, the Porter laboratory expands on their previous work and explores the role of neonatal hypoxia on mitochondrial permeability transition and cardiac maturation.^6^ The PO2 in the early embryo (around E8.5 in the mouse heart) is lower than the rest of the embryo and is believed to keep cardiac myocytes in an immature and proliferative state before and during cardiac looping. However, increased oxygenation at birth reverses this phenomenon to promote cardiac and mitochondrial maturation. My laboratory, and others, have shown that neonatal hypoxia can sustain cardiomyocyte proliferation, can enhance glycolytic metabolism, and ultimately, can lead to cardiomyocyte necrosis.^7^^,^^8^ The new work by the Porter laboratory extends the translational significance of these findings and further explores the cardiac phenotype elicited by neonatal hypoxia using both murine and larger-mammal lamb models. Neonatal hypoxia is a common complication of numerous perinatal pathologies including placental abnormalities, preterm birth, and asphyxia, as well as congenital defects affecting the heart and lung. This new work demonstrates that cardiac structure and contractile function are disrupted through enhanced proliferation and altered mitochondrial bioenergetics. The bioenergetics defect was traced to complex I, and to a lesser extent, complex II. Importantly, all of these defects were prevented by inhibiting cyclophilin D with either cyclosporin A or NIM811, suggesting that mitochondrial permeability transition regulates cardiac mass through myocyte proliferation, while improving ejection fraction by enhancing mitochondrial and contractile function.

In addition to the translational importance, the strength of these studies lies within the detailed bioenergetics and interrogation of the mitochondrial electron transport chain (ETC) complexes. Hypoxia did affect expression of individual ETC genes, proteins, or complex monomers, but did not alter mitochondrial biogenesis. Hypoxia was detrimental to mitochondrial complex I and II activity, and also increased ROS production. These observations suggest some form of mitochondrial uncoupling that can be reversed by PTP inhibition. However, no gross mitochondrial ultrastructure changes were observed in this study. Thus, the alteration in complex I and II activity could be allosteric or related to spatial-temporal rearrangements of complexes within the inner mitochondrial membrane that are sensitive to PTP closure, and can result in more efficient transfer of electrons. Clearly, additional work is needed to explain how PTP closure affects ETC function.

This study is not without limitations, as outlined by the authors in their discussion. First, nonmyocyte cells of the heart would have been affected by both hypoxia and the cyclophilin D inhibitors, which could indirectly influence contractile function. However, histological analysis did not reveal fibrosis or other phenotypes related to nonmyocytes. It is also unlikely that nonmyocytes biased the bioenergetic analysis. Finally, sex differences were not considered in the current study, and the authors concede that this is an important limitation that should be rectified with further work.

In conclusion, the Porter laboratory now demonstrates that neonatal hypoxia has detrimental effects on cardiac function, and that changes in mitochondrial bioenergetics likely disrupts the dynamic balance of myocyte proliferation and differentiation. In addition, hypoxia-induced changes in cardiac function are rescued by inhibition of the PTP and have the potential to restore the normal growth trajectory of the neonatal heart. Thus, targeting cyclophilin D, either directly or indirectly, may prove to be therapeutic for neonatal cardiac dysfunction and the prevention of cardiomyopathy later in life.

## Funding Support and Author Disclosures

Dr Gordon has reported that he has no relationships relevant to the contents of this paper to disclose.
